# Umbilical cord/placenta-derived mesenchymal stem cells inhibit fibrogenic activation in human intestinal myofibroblasts via inhibition of myocardin-related transcription factor A

**DOI:** 10.1186/s13287-019-1385-8

**Published:** 2019-09-23

**Authors:** Yoon Jeong Choi, Jun Bon Koo, Hee Yeon Kim, Jin Won Seo, Eun Jeong Lee, Woo Ram Kim, Joo Young Cho, Ki Baik Hahm, Sung Pyo Hong, Duk Hwan Kim, Jun-Hwan Yoo

**Affiliations:** 1 Digestive Disease Center, CHA Bundang Medical Center, CHA University, 59 Yatap-ro, Bundang-gu, Seongnam, 463-712 South Korea; 2 Institute of Basic Medical Sciences, School of Medicine, CHA University, Seongnam, South Korea; 3 Clinical Research Center, CHA Bundang Medical Center, CHA University, Seongnam, South Korea; 4 CHA Biotech, Co. Ltd., Seongnam, South Korea; 5 Department of Surgery, CHA Bundang Medical Center, CHA University, Seongnam, South Korea

**Keywords:** Intestinal fibrosis, Myofibroblasts, Mesenchymal stem cells, Umbilical cord, Placenta

## Abstract

**Background:**

The lack of anti-fibrotic agents targeting intestinal fibrosis is a large unmet need in inflammatory bowel diseases, including Crohn’s disease and ulcerative colitis. Previous studies have found that perinatal tissue (umbilical cord, UC; placenta, PL)-derived mesenchymal stem cells (MSCs) reduce fibrosis in several organs. However, their effects on human intestinal fibrosis are poorly understood. This study investigated the anti-fibrogenic properties and mechanisms of MSCs derived from UC and PL (UC/PL-MSCs) on human primary intestinal myofibroblasts (HIMFs).

**Methods:**

The HIMFs were treated with TGF-β1 and co-cultured with UC/PL-MSCs. We used a small molecular inhibitor CCG-100602 to examine whether serum response factor (SRF) and its transcriptional cofactor myocardin-related transcription factor A (MRTF-A) are involved in TGF-β1-induced fibrogenic activation in HIMFs. The anti-fibrogenic mechanism of UC/PL-MSCs on HIMFs was analyzed by detecting the expression of RhoA, MRTF-A, and SRF in HIMFs.

**Results:**

UC/PL-MSCs reduced TGF-β1-induced procollagen1A1, fibronectin, and α-smooth muscle actin expression in HIMFs. This anti-fibrogenic effect was more apparent in the UC-MSCs. TGF-β1 stimulation increased the expressions of RhoA, MRTF-A, and SRF in the HIMFs. TGF-β1 induced the synthesis of procollagen1A1, fibronectin, and α-smooth muscle actin through a MRTF-A/SRF-dependent mechanism. Co-culture with the UC/PL-MSCs downregulated fibrogenesis by inhibition of RhoA, MRTF-A, and SRF expression.

**Conclusions:**

UC/PL-MSCs suppress TGF-β1-induced fibrogenic activation in HIMFs by blocking the Rho/MRTF/SRF pathway and could be considered as a novel candidate for stem cell-based therapy of intestinal fibrosis.

**Electronic supplementary material:**

The online version of this article (10.1186/s13287-019-1385-8) contains supplementary material, which is available to authorized users.

## Background

Intestinal fibrosis is a serious complication of inflammatory bowel diseases (IBD), which is clinically more apparent in Crohn’s disease (CD). Up to one third of CD patients will develop an end-stage fibrotic disease characterized by intestinal strictures leading to obstruction and necessitating surgery [1, 2]. After surgical resection, up to 70% of CD patients will suffer recurrent endoscopic disease at 1 year [2]. While current anti-inflammatory therapies may relieve inflammatory strictures, fibrostenotic strictures are not resolved by immunosuppressive therapies [3]. Although intestinal fibrosis is initiated by recurrent inflammation, elimination of inflammation alone does not prevent or reverse established fibrosis, suggesting that the development of direct anti-fibrotic therapy approaches is necessary [4].

Fibrosis is characterized by excessive deposition of extracellular matrix (ECM) proteins including collagen and fibronectin, which are synthesized by activated myofibroblasts [5]. Activated myofibroblasts express elevated levels of α-smooth muscle actin (α-SMA) and consequently exert a markedly enhanced capability to contract the ECM, contributing to tissue distortion and intestinal strictures [6]. Transforming growth factor-beta (TGF-β) has been recognized as a pivotal pro-fibrotic cytokine to induce fibrogenic activation of myofibroblasts [7, 8]. TGF-β-induced fibrogenic activation in myofibroblasts is mediated by the Smad-dependent and Smad-independent TGF-β signaling pathways [9–12]. The Smad-dependent TGF-β signaling is transduced by phosphorylation of Smad2 and Smad3, which translocate into the nucleus and induce the transcription of pro-fibrotic genes [9, 11, 13]. The Smad-independent TGF-β signaling is transduced by phosphorylation of extracellular signal-regulated kinase (ERK), c-Jun N-terminal kinase (JNK), p38 mitogen-activated protein kinase (MAPK), and AKT [8, 11, 12, 14]. Another Smad-independent TGF-β signaling includes Ras homolog family member A/Rho-associated coiled-coil forming protein kinase (RhoA/ROCK) pathway. RhoA-mediated ROCK activation results in polymerization of globular-actin (G-actin) into filamentous actin (F-actin) and releases transcriptional cofactor myocardin-related transcription factor A (MRTF-A) from the G-actin, enabling it to translocate into the nucleus and bind to the pro-fibrotic transcription factor, serum response factor (SRF) [10, 15].

Previous clinical trials have reported that the infusion of mesenchymal stem cells (MSCs) has anti-fibrotic potential in several diseases including decompensated liver cirrhosis [16, 17], renal interstitial fibrosis due to allograft rejection [18], and ischemic cardiomyopathy [19, 20]. Preclinical studies have also demonstrated that MSCs can attenuate fibrosis in several organs (liver, lungs, kidney, heart, skin, peritoneum, pancreas, and colorectum) [21] by several mechanisms such as releasing anti-fibrotic molecules (hepatocyte growth factor [22] and TNF-stimulated gene 6 [23]), inhibiting TGF-β activation by reducing oxidative stress (reactive oxygen and nitrogen species) [21, 24], and ECM remodeling [25]. Most previous studies using MSCs have used the bone marrow (BM) and adipose tissue (AT) as major cell sources [26]. However, there are several disadvantages in using BM/AT-MSCs due to the invasive procedure and the significant decrease in cell number and proliferation/differentiation capacity with the increasing age of the donor [27]. In contrast to the BM/AT-MSCs, umbilical cord/placenta-derived mesenchymal stem cells (UC/PL-MSCs) can be obtained in larger quantities without an invasive procedure and have a greater proliferation/differentiation potency, representing a promising alternative MSC population for clinical applications [26, 28, 29].

Although previous studies have shown the anti-fibrotic effect of MSCs on several organs, the potential of human UC/PL-MSCs to reduce intestinal fibrosis has not yet been evaluated to our knowledge. Moreover, compared to the number of in vivo studies showing that MSC therapy ameliorates organ fibrosis, the number of in vitro studies that revealed the specific intracellular anti-fibrotic mechanism of MSCs is still limited. This study was therefore designed to determine whether UC/PL-MSCs have an inhibitory effect on the fibrogenic activation of human intestinal myofibroblasts (HIMFs) and to determine the intracellular mechanisms involved with the inhibitory effect.

## Methods

### Reagents

Recombinant human TGF-β1 was obtained from R&D systems (Minneapolis, MN). CCG-100602 (1-[3,5-bis (trifluoromethyl)benzoyl]-*N*-(4-chlorophenyl)-3-piperidinecarboxamide) was purchased from Cayman Chemical (Ann Arbor, MI).

### Human intestinal myofibroblast isolation and culture

Primary HIMFs were isolated and cultured with some modifications as previously described [30]. Briefly, HIMFs were derived from outgrowths of minced colonic mucosa explants placed on etched polystyrene flasks containing HIMFs growth medium consisting of Dulbecco’s modified Eagle’s medium/high glucose (Hyclone, Logan, UT), 10% fetal bovine serum (American Type Culture Collection, Manassas, VA), 4 mmol/L l-glutamine (Gibco, Carlsbad, CA), 25 mmol/L HEPES, 100 U/mL penicillin, 100 μg/mL streptomycin, and 0.25 μg/mL amphotericin B (all purchased from Lonza, Walkersville, MD) and used between passages 6 and 10 at 80% confluence. HIMFs were isolated from normal colon segments of patients undergoing resection due to colorectal cancer. The grossly normal colon segments were taken from the area, which was near the proximal resection margin, by a pathologist after surgical resection. The periphery of the normal colon segments, which was used for isolation, was histologically confirmed through a microscope. The project was performed in accordance with the guidelines of the Institutional Review Board of the CHA Bundang Medical Center.

### Preparation of human UC/PL-MSCs

Human umbilical cord- and placenta-derived MSCs were provided by CHA Biotech, Co. Ltd. (Seongnam, Korea). Preparations of the human UC/PL-MSCs were conducted in the Good manufacturing practices (GMP) facility, and the isolation and expansion of human UC/PL-MSCs were performed according to the Good Clinical Practice (GCP) guidelines of the Master Cell Bank. Preparation and characterization of the cells have been described previously [31–34]. Umbilical cord and placenta tissue were obtained with informed consent from healthy mother donors at CHA Bundang Medical Center (Seongnam, Korea). To isolate UC-MSCs, Wharton’s jelly was sliced into 1–5-mm explants after the umbilical vessels were removed. Isolated slices were attached on culture plates and subsequently cultured in α-modified minimal essential medium (α-MEM; Hyclone) supplemented with 10% fetal bovine serum (FBS; Gibco), 25 ng/mL fibroblast growth factor-4 (FGF4; Peprotech, London, England), 1 μg/mL heparin (Sigma, St. Louis, MO), and 0.5% gentamycin (Gibco) at 37 °C in a humidified atmosphere containing 5% CO_2_. The medium was changed every 3 days, and the UC-MSC cell populations appeared as outgrowths from the UC fragments at day 6. After 15 days, the umbilical cord fragments were discarded, and the cells were passaged with TrypLE (Invitrogen, Carlsbad, CA) and expanded until they reached sub-confluence (80–90%). Fluorescence-activated cell sorting (FACS) analysis was used to identify the phenotype of the cells, and UC-MSCs at passage 6 were used in the present study.

To isolate the PL-MSCs, the placental membranes were separated by blunt dissection from the placental body and washed in Dulbecco’s phosphate-buffered saline (DPBS; Gibco) to remove the blood. Amniotic connective tissue of the placental membranes was carefully harvested by using two slide glasses and then incubated at 37 °C with shaking (175 rpm) for 15 min with HBSS containing 1 mg/mL type I collagenase (Sigma), 1.2 U/mL dispase (Gibco), 2 mg/mL trypsin (Sigma), 65 μg/mL DNase I (Roche, Mannheim, Germany), and 1× penicillin-streptomycin (Gibco). The viability of the isolated cells was determined by trypan blue exclusion. PL-MSCs were cultured in α-MEM (Hyclone) supplemented with 10% FBS (Gibco), 25 ng/mL FGF4 (Peprotech), 1 μg/mL heparin (Sigma), and 0.5% gentamycin (Gibco) at 37 °C in a humidified atmosphere containing 5% CO_2_. FACS analysis was used to identify the phenotype of the cells, and the PL-MSCs at passage 6 were used in the present study.

### Co-culture of HIMFs with UC/PL-MSCs

A co-culture transwell chamber (24-mm diameter, 3.0-μm pore size; Corning, NY) was used to assess the effects of UC/PL-MSCs on HIMFs in vitro. Thus, 1.0 × 10^5^ or 2.0 × 10^5^ UC/PL-MSCs were seeded onto the transwell insert while 1.5 × 10^5^ HIMFs were cultured at the bottom of the 6-well plate. This system enables the apical surface of the HIMFs to be exposed to the soluble factors secreted by the UC/PL-MSCs. To evaluate the anti-fibrogenic properties of the UC/PL-MSCs, the HIMFs (1.5 × 10^5^ cells/well) were starved for 24 h and then treated with 5 ng/mL TGF-β1 alone or co-cultured with UC/PL-MSCs in serum-free HIMF culture medium. After 48 h, HIMF RNA extracts and proteins from the lower chamber were examined by real-time RT-PCR and Western blot, respectively.

### RNA isolation and qRT-PCR

Total RNA was extracted from the HIMFs using the TRIzol reagent (Ambion, Carlsbad, CA), and then, an equal amount of RNA (1 μg) was reverse-transcribed into cDNA using the ReverTra Ace qPCR RT Master Mix Kit (TOYOBO, Osaka, Japan) according to the manufacturer’s instructions. All qRT-PCR reactions were performed using a Roche Light Cycler 96 instrument (Roche) with FasterStart Essential DNA Probes Master (Roche). The mRNA levels of all genes were normalized to that of GAPDH. The specific primers for the collagen1A1 (Hs00164004_m1), fibronectin (Hs01549976_m1), α-SMA (ACTA2, Hs00426835_g1), Mkl1 (MRTF-A, Hs01090249_g1), SRF (Hs01065256_m1), RhoA (Hs00357608_m1), Rock1 (Hs01127701_m1), Rock2 (Hs00178154_m1) and GAPDH (Hs03929097_g1) genes were purchased from Applied Biosystems (Foster City, CA).

### Western blot

Protein extracts were isolated using RIPA buffer (Cell Signaling, Beverly, MA). Protein samples were mixed with an equal volume of 5 × SDS sample buffer, boiled for 5 min, and then separated on 10% SDS-PAGE gels. After electrophoresis, the proteins were transferred to polyvinylidene difluoride membranes. The membranes were blocked with 5% nonfat dry milk in Tris-buffered saline with Tween-20 buffer (TBS-T) for 1 h at room temperature. Membranes were incubated overnight at 4 °C with specific antibodies. Primary antibodies were removed by washing the membranes three times in TBS-T and incubated for 2 h with horseradish peroxidase-conjugated anti-rabbit or anti-mouse immunoglobulin (GeneTex, Irvine, CA). Following three washes with TBS-T, antigen-antibody complexes were detected using the SuperSignal West Pico Chemiluminescence System (Thermo Fisher Scientific, Rockford, IL). The signals were captured with a luminescent image analyzer (ChemiDocTM XRS+ System, Bio-Rad, USA). The quantification of the Western blots was performed using the ImageJ 1.50i software (Wayne Rasband, National Institute of Health, USA). The antibodies used were as follows: procollagen1A1 (Procol1A1) antibody (SP1D8, Developmental Studies Hybridoma Bank, Iowa City, IA), fibronectin (FN) antibody (ab2413, Abcam, Cambridge, MA), α-smooth muscle actin (α-SMA) antibody (A2547, Sigma), phospho-Smad2 (Ser465/467) antibody (#3108, Cell Signaling), phospho-Smad3 antibody (#07-1389, Millipore, Temecula, CA), phospho-ERK antibody (#9101, Cell Signaling), phospho-JNK antibody (#4668, Cell Signaling), phospho-p38 MAPK antibody (#4511, Cell Signaling), phospho-AKT antibody (#9271, Cell Signaling), RhoA antibody (#sc-418, Santa Cruz Biotechnology, Dallas, TX), GAPDH antibody (#2118, Cell Signaling), Mkl1(MRTF-A) antibody (ab49311, Abcam), SRF antibody (#5147, Cell Signaling), and HDAC1 antibody (#5356, Cell Signaling).

### Immunocytochemistry

Immunofluorescence staining was performed as previously described [8]. To evaluate the anti-fibrogenic properties of the UC/PL-MSCs, the HIMFs (1.5 × 10^5^ cells/well) were seeded onto a 6-well plate and exposed to TGF-β1 (5 ng/mL) co-cultured with or without the UC/PL-MSCs (2.0 × 10^5^/insert) for 48 h. Before seeding the HIMFs, sterilized microscope cover glasses were placed into the wells of a 6-well plate for immunostaining. To examine the effect of CCG-100602, the HIMFs (1.0 × 10^4^ cells/well) were seeded onto the chamber slides (#30108, SPL life Sciences, Korea) and exposed to TGF-β1 (5 ng/mL) co-incubated with or without CCG-100602 (40 μmol/L) for 48 h after which the cells were fixed with 4% paraformaldehyde (PFA) for 10 min. After permeabilization with 0.1% Triton X-100 in 1 × PBS and blocking with 5% bovine serum albumin (BSA), the cells were incubated with primary antibodies at 4 °C overnight. All antibodies used for immunocytochemistry are described in the *Western blot*. After washing three times with PBS (10 min each), the cells were incubated with AlexaFluor488/594-conjugated goat anti-mouse/rabbit secondary antibody (Molecular Probes, Eugene, OR) at room temperature for 2 h. For the staining of the actin filaments (F-actin), the cells were incubated with rhodamine-phalloidin (1:500, R415, Invitrogen) at room temperature for 30 min. After washing, the nuclei were counterstained by Hoechst33342 (B2261, Sigma) according to the manufacturer’s instructions. Images were acquired using a Zeiss LSM880 confocal laser scanning microscope. To quantify the nuclear-to-cytoplasmic ratio, images were imported into the ImageJ 1.50i software. Using the Cell Mask stain, individual cells were outlined, and the optical density of the MRTF-A staining was measured and adjusted for the area of the cell. Next, the Hoechst33342 stain was used to similarly outline the nucleus and calculate the density of the MRTF-A staining within the nucleus. The cytoplasmic fraction was determined by subtracting the nuclear fraction from the total cell calculation, and the nuclear-to-cytoplasmic ratio was determined by dividing the nuclear signal by the cytoplasmic signal.

### Extraction of nuclear and cytoplasmic proteins

Nuclear and cytoplasmic fractions of the HIMFs were obtained using the Nuclear Extraction kit (Millipore) according to the manufacturer’s protocol. The protein concentration was measured by the bicinchoninic acid (BCA) protein assay, and Western blotting was performed.

### Statistical analysis

Data are expressed as the mean ± SEM (standard error of the mean) of at least three independent experiments. Comparison between two groups was performed using the Mann-Whitney *U* test. For multiple comparisons, analysis of variance (ANOVA) was used with Tukey’s post hoc test. *P* values less than 0.05 were considered statistically significant. All statistical analyses were performed using GraphPad Prism 5.0 (GraphPad, San Diego, CA, USA).

## Results

### Characterization of UC/PL-MSCs

The morphologies of UC- and PL-MSCs were similar to the round-spindle shape of mesenchymal stem cells (Additional file 1: Figure S1A and B). To identify the surface phenotype of the UC/PL-MSCs, we performed FACS analysis. The expression of CD44, CD73, and CD105 as well as the lack of CD45, CD34, and CD31 were identified on the cells which were isolated and cultured as UC- and PL-MSCs (Additional file 1: Figure S1C and D).

### UC/PL-MSCs inhibit TGF-β1-induced ECM and α-SMA expression in human intestinal myofibroblasts

**Fig. 1:**
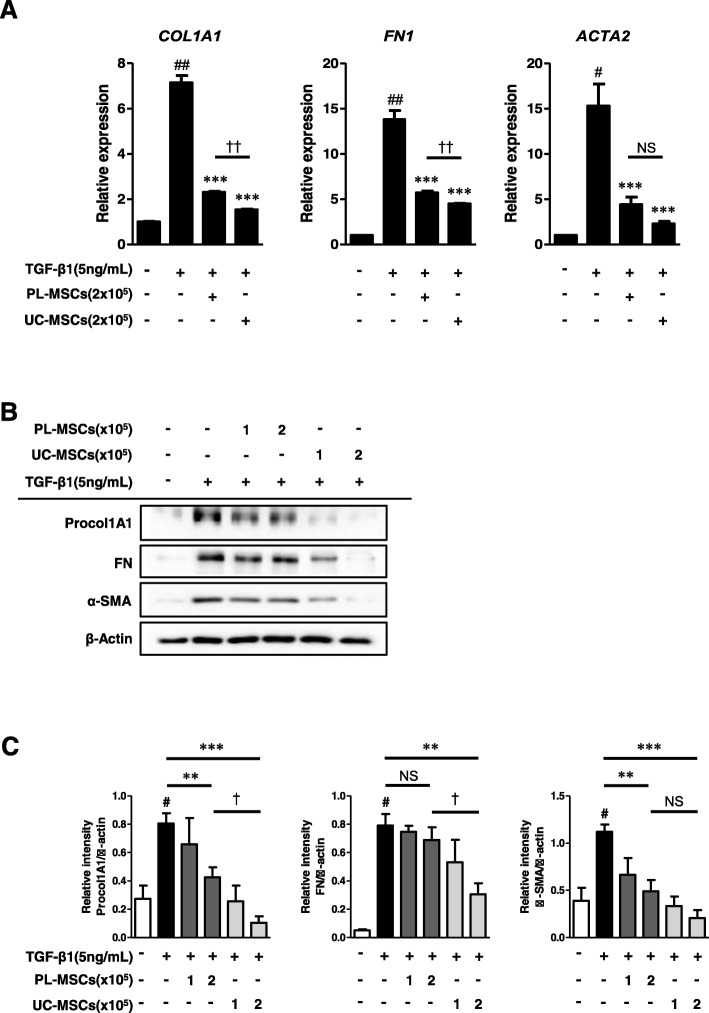
Co-culture with umbilical cord/placenta-derived mesenchymal stem cells (UC/PL-MSCs) inhibits TGF-β1-induced fibrogenic activation of human intestinal myofibroblasts (HIMFs). HIMFs were treated with TGF-β1 (5 ng/mL) and co-cultured with or without UC/PL-MSCs at 1 or 2 × 10^5^ cells/insert for 48 h. **a** qPCR analysis of the relative mRNA expression of collagen1A1 (*COL1A1*), fibronectin (*FN1*), and α-smooth muscle actin (*ACTA2*). The data were normalized to GAPDH expression and expressed as relative values compared with the control (*n* = 3). UC/PL-MSCs were seeded at 2 × 10^5^ cells/insert. **b** Representative Western blots show the protein expression of procollagen1A1 (Procol1A1), fibronectin (FN), and α-smooth muscle actin (α-SMA) with β-Actin as a loading control. **c** Quantitation of Procol1A1, FN, and α-SMA from Western blot analyses (*n* = 4). Data are expressed as the means ± SEM. ^#^*P* < 0.05 and ^##^*P* < 0.01 versus the untreated control; **P < 0.01 and ***P < 0.001 versus the TGF-β1 treatment only; ^†^*P* < 0.05 and ^††^*P* < 0.01 compared between the UC-MSCs and PL-MSCs co-culture. NS, not significant

To determine whether UC/PL-MSCs inhibit fibrogenic activation of myofibroblasts, HIMFs were co-cultured with UC/PL-MSCs and simultaneously stimulated with TGF-β1. As shown in Fig. 1a, TGF-β1 markedly increased the mRNA expression of collagen1A1 (*COL1A1*), FN (*FN1*), and α-SMA (*ACTA2*) in the HIMFs, and co-culture with UC- or PL-MSCs abolished this effect. The reduction of *COL1A1* and *FN1* mRNA expression was more prominent in the UC-MSCs than in the PL-MSCs. Although there was no significant difference (*P* = 0.22) in the reduction of the *ACTA2* expression between the UC- and PL-MSCs, the UC-MSCs showed a more reductive trend compared to the PL-MSCs.

The anti-fibrotic effect of the UC- and PL-MSCs was also identified at the protein level. As shown in Fig. 1b, c, the TGF-β1-induced upregulation in the protein expression of Procol1A1 and α-SMA was significantly reduced by high numbers (2 × 10^5^ cells) of UC- and PL-MSCs. UC-MSCs, not PL-MSCs, also significantly decreased the TGF-β1-induced upregulation in FN protein expression. Consistent with the results at the mRNA level, the UC-MSCs were more potent than the PL-MSCs in the reduction of ECM protein synthesis (Procol1A1 and FN) in the HIMFs. However, there was no significant difference (*P* = 0.11) in the reduction of α-SMA synthesis, despite a more reductive trend in the UC-MSCs, between the UC- and PL-MSCs.

**Fig. 2:**
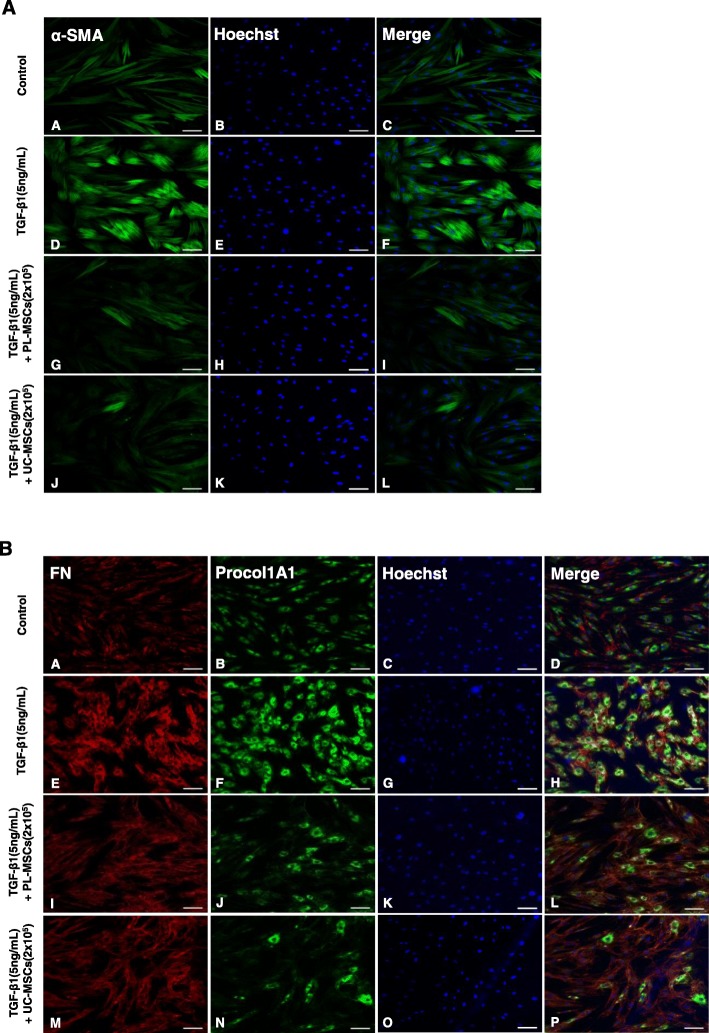
Co-culture with UC/PL-MSCs inhibits TGF-β1-induced Procol1A1, FN, and α-SMA expression in HIMFs. HIMFs were treated with TGF-β1 (5 ng/mL) and co-cultured with or without UC/PL-MSCs at 2 × 10^5^ cells/insert for 48 h and then stained with Procol1A1, FN, and α-SMA antibodies and counterstained with Hoechst. **a** α-SMA. **b** Procol1A1 and FN. Scale bars, 100 μm; original magnification, × 100

Increased α-SMA expression and the subsequent assembly of α-SMA into stress fibers, which are formed by F-actin polymerization, are hallmarks of activated myofibroblasts [35]. In TGF-β1-stimulated cells, α-SMA immunostaining showed well-organized intensely stained actin stress fibers. In contrast, cells stimulated with TGF-β1 in the presence of the UC- or PL-MSCs exhibited a diffuse, muted α-SMA staining with a lack of organized stress fibers similar to the α-SMA staining observed in the control (Fig. 2a). Immunostaining of Procol1A1 and FN also showed that co-culture with the UC/PL-MSCs diminished the TGF-β1-enhanced staining of Procol1A1 and FN in the HIMFs (Fig. 2b). Collectively, these data suggest that the UC and PL-MSCs inhibit TGF-β1-induced fibrogenic activation of the HIMFs by downregulating the ECM and α-SMA at both the mRNA and protein levels.

### TGF-β1 induces myofibroblast activation by MRTF-A/SRF-dependent signaling

**Fig. 3:**
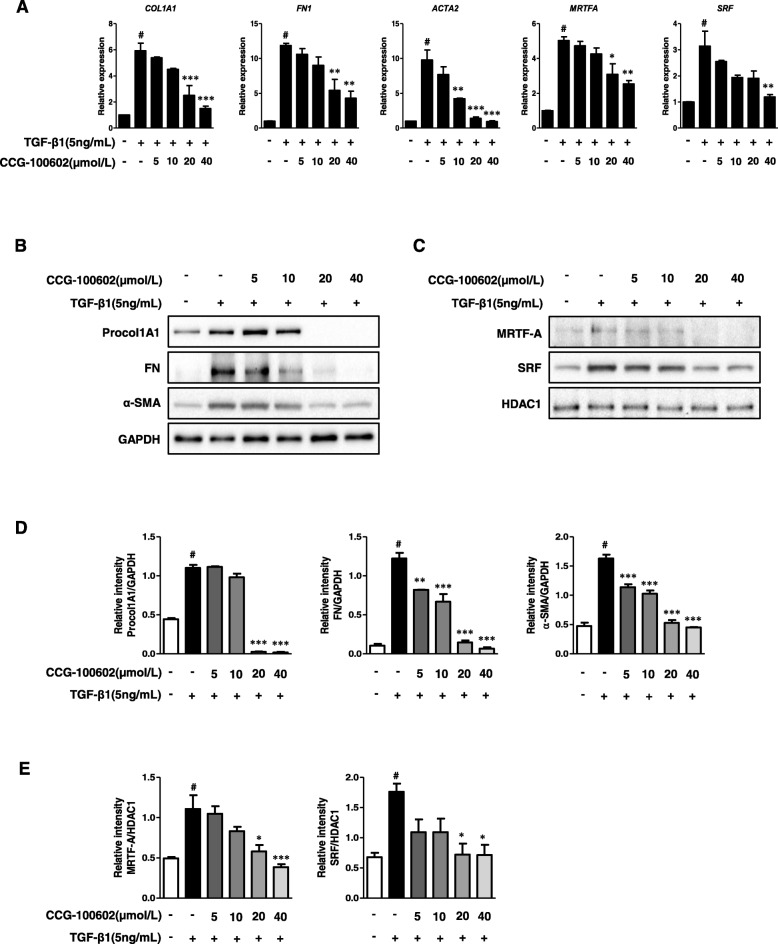
TGF-β1-induced fibrogenic activation of HIMFs is MRTF-A/SRF dependent. HIMFs were pretreated with or without CCG-100602 at 5, 10, 20, and 40 μmol/L concentrations for 30 min prior to the addition of TGF-β1 (5 ng/mL) for 24 (qPCR) or 48 (Western blots) hours. **a** qPCR analysis of the relative mRNA expression of *COL1A1*, *FN1*, *ACTA2*, *MRTFA*, and *SRF*. The data were normalized to the GAPDH expression and expressed as relative values compared with the control (*n* = 3). **b** Representative Western blots show the protein expression of Procol1A1, FN, and α-SMA with GAPDH as a loading control. **c** Representative Western blots show the protein expression of MRTF-A and SRF in the nuclear extracts with histone deacetylase 1 (HDAC1) as a loading control of the nuclear fraction. **d**, **e** Quantitation of Procol1A1, FN, α-SMA, MRTF-A, and SRF from the Western blot analyses (*n* = 3). Data are expressed as the means ± SEM. ^#^*P* < 0.05 versus the untreated control; *P < 0.05, **P < 0.01, and ***P < 0.001 versus the TGF-β1 treatment only

A previous study demonstrated that TGF-β-induced fibrogenesis in the human colonic myofibroblast cell line (CCD-18co) was associated with MRTF-A nuclear localization. The small molecule inhibitors (CCG-100602, CCG-203971), which specifically block MRTF-A nuclear localization and thus inhibit the fibrogenic transcription factor SRF, lead to disruption of the TGF-β-mediated mRNA expression of *COL1A1*, *ACTA2*, and *MRTFA* [10]. To test whether MRTF-A/SRF signaling is involved in the TGF-β1-induced ECM and α-SMA expression in the HIMFs, we used CCG-100602 as a specific inhibitor of MRTF-A/SRF signaling. As expected, CCG-100602 diminished the TGF-β1-induced increase in *COL1A1*, *FN1*, and *ACTA2* transcription in a dose-dependent manner. Moreover, CCG-100602 reduced the TGF-β1-induced increase in *MRTFA* and *SRF* mRNA expression in the HIMFs in a dose-dependent manner (Fig. 3a).

Consistent with the alteration of the mRNA expression, the protein expression levels of the ECM and α-SMA in TGF-β1-stimulated cells were significantly reduced by the CCG-100602 treatment in a dose-dependent manner (Fig. 3b, d). CCG-100602 also significantly repressed the MRTF-A and SRF protein expression, which were induced by TGF-β1, in the nuclear fraction of the HIMFs in a dose-responsive manner (Fig. 3c, e).

**Fig. 4:**
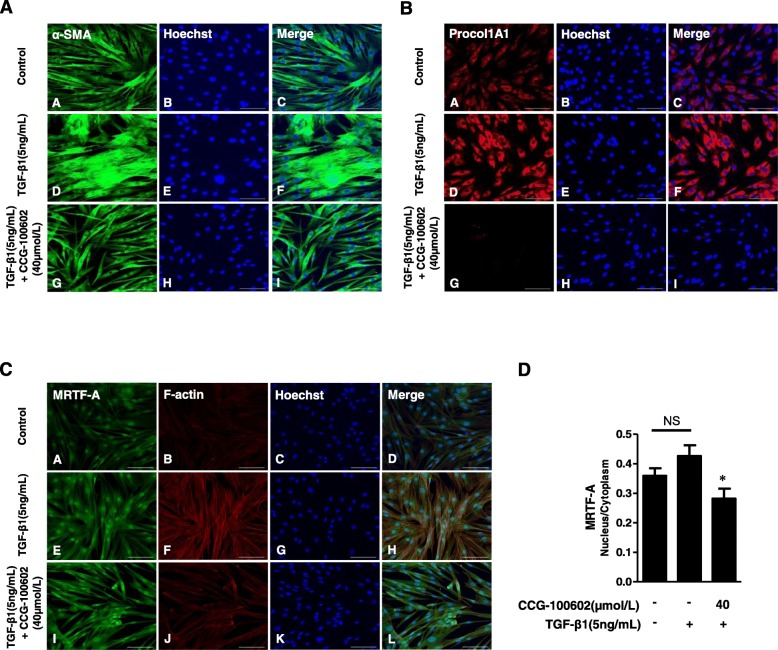
TGF-β1 induces Procol1A1 and α-SMA expression by MRTF-A/SRF-dependent signaling in HIMFs. HIMFs were pretreated with or without CCG-100602 (40 μmol/L) for 30 min prior to the addition of TGF-β1 (5 ng/mL) for 48 h and then stained with Procol1A1, α-SMA, and MRTF-A antibodies and rhodamine-phalloidin and counterstained with Hoechst. **a** α-SMA. **b** Procol1A1. **c** MRTF-A and F-actin. **d** The nuclear-to-cytoplasmic ratio of MRTF-A was determined for each condition as described in the the “Methods” section. Data are expressed as the means ± SEM. *P < 0.05 versus the TGF-β1 treatment only. NS, not significant. Scale bars, 100 μm; original magnification, × 200 (**a**–**c**)

In the immunostaining of α-SMA, compared with the well-organized, intensely stained stress fibers in the HIMFs stimulated with TGF-β1, the cells co-treated with CCG-100602 exhibited less organized stress fibers with reduced α-SMA staining (Fig. 4a). The intensity of Procol1A1 staining enhanced with TGF-β1 in the HIMFs was markedly reduced by the co-treatment with CCG-100602 (Fig. 4b). To further investigate whether the nuclear translocation of MRTF-A was effectively blocked by CCG-100602, as demonstrated in a colonic myofibroblast cell line (CCD-18co) by a previous study [10], the subcellular localization of MRTF-A was determined by immunocytochemistry, and the nuclear-to-cytoplasmic ratio was quantified. The release and nuclear translocation of MRTF-A is induced by G-actin depletion following F-actin polymerization. Therefore, we further stained F-actin with rhodamine-phalloidin. MRTF-A nuclear localization was not statistically different between untreated cells and TGF-β1-treated cell. However, F-actin formation was significantly increased by the TGF-β1 treatment. CCG-100602 led to a significant reduction in MRTF-A nuclear localization and F-actin formation in the TGF-β1-treated myofibroblasts (Fig. 4c, d). These data are consistent with the previous study [10]. Taken together, these data suggest that MRTF-A/SRF signaling is implicated in the TGF-β1-induced ECM and α-SMA expression in the HIMFs.

### UC/PL-MSCs inhibit the Rho/MRTF/SRF signaling in myofibroblasts

**Fig. 5:**
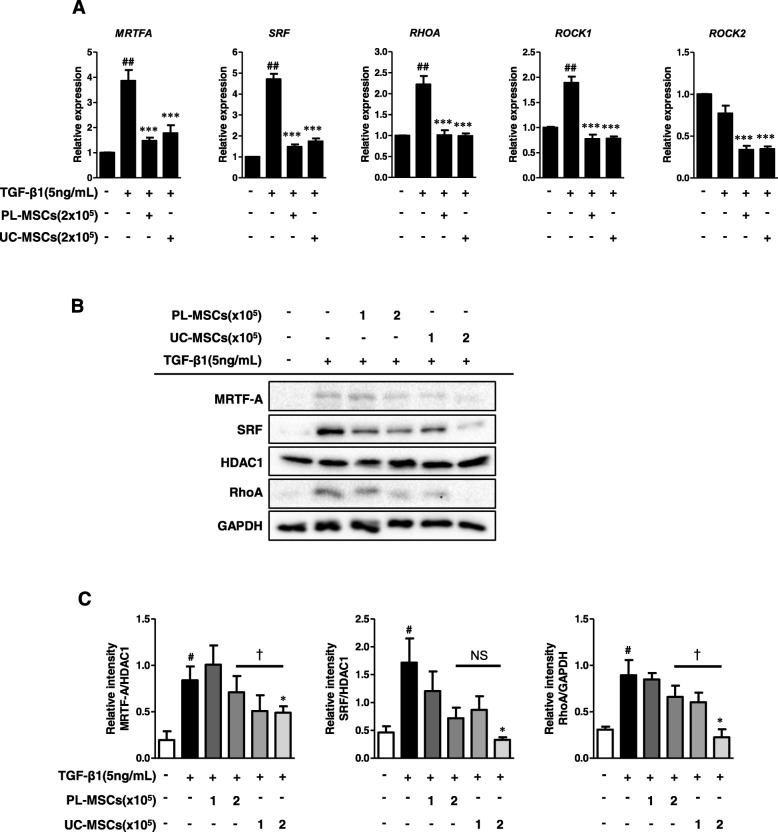
UC/PL-MSCs inhibit Rho/MRTF/SRF signaling in HIMFs. HIMFs were treated with TGF-β1 (5 ng/mL) and co-cultured with or without UC/PL-MSCs at 1 or 2 × 10^5^ cells/insert for 48 h. **a** qPCR analysis of the relative mRNA expression of *MRTFA*, *SRF*, *RHOA*, *ROCK1*, and *ROCK2*. The data were normalized to GAPDH expression and expressed as relative values compared with the control (*n* = 3). **b** Representative Western blots show the protein expression of MRTF-A, SRF, and RhoA (MRTF-A and SRF from the nuclear extracts with HDAC1 as a loading control, RhoA from the cytosolic extracts with GAPDH as a loading control). **c** Quantitation of MRTF-A, SRF, and RhoA from Western blot analyses (*n* = 3). Data are expressed as the means ± SEM. ^#^*P* < 0.05 and ^##^*P* < 0.01 versus the untreated control; *P < 0.05 and ***P < 0.001 versus the TGF-β1 treatment only; ^†^*P* < 0.05 compared between the UC-MSCs and PL-MSCs co-culture. NS, not significant

**Fig. 6:**
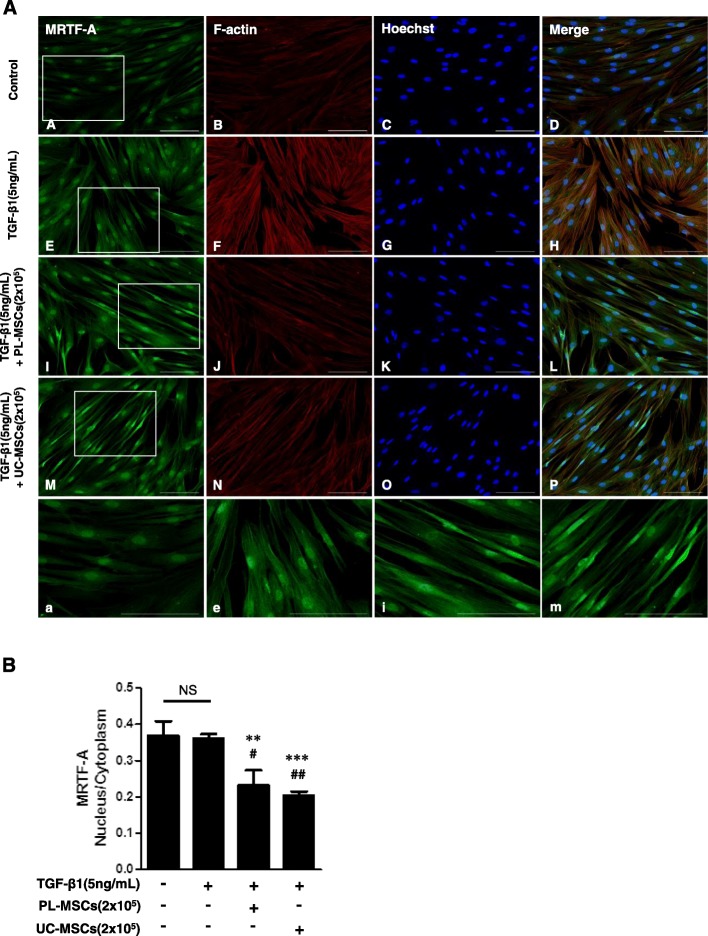
UC/PL-MSCs inhibit MRTF-A nuclear localization in HIMFs. HIMFs were treated with TGF-β1 (5 ng/mL) and co-cultured with or without UC/PL-MSCs at 2 × 10^5^ cells/insert for 48 h and then stained with MRTF-A antibodies and rhodamine-phalloidin and counterstained with Hoechst. **A** (A–D) no treatment; (E–H) treatment with TGF-β1; **(**I-L) treatment with TGF-β1 and PL-MSCs; (M–P) treatment with TGF-β1 and UC-MSCs. a, e, i, m Enlarged images of the region (within the white box of A, E, I, and M, each). **B** The nuclear-to-cytoplasmic ratio of MRTF-A was determined for each condition as described in the “Methods” section. Data are expressed as the means ± SEM. ^#^*P* < 0.05 and ^##^*P* < 0.01 versus the untreated control; **P < 0.01 and ***P < 0.001 versus the TGF-β1 treatment only; NS, not significant. Scale bars, 100 μm; original magnification, × 200 (**A**)

Our next question was whether UC/PL-MSCs reduced the TGF-β1-induced Procol1A1, FN, and α-SMA expression by inhibiting the MRTF/SRF signaling in the HIMFs. As shown in Fig. 5a, UC and PL-MSCs significantly decreased the TGF-β1-induced *MRTFA* and *SRF* mRNA expression. We further evaluated the expression of upstream signaling molecules, including RhoA, ROCK1, and ROCK2, which contribute to F-actin polymerization upstream of the MRTF/SRF pathway (Rho/ROCK/Actin/MRTF/SRF Axis [15]). In the HIMFs, the UC/PL-MSCs diminished the mRNA expression levels of *RHOA* and *ROCK1* induced by TGF-β1. The *ROCK2* mRNA expression, which was not induced by TGF-β1, was significantly reduced in the UC/PL-MSC co-culture compared with the TGF-β1 treatment alone (Fig. 5a). At the protein level, high numbers (2 × 10^5^ cells) of UC-MSCs significantly reduced the MRTF-A and SRF expressions in the nuclear extracts and the RhoA expression in the cytosolic extracts, which were induced by TGF-β1. Though not statistically significant, high numbers of PL-MSCs also showed a reductive trend, especially in SRF expression with a modest 50% reduction (Fig. 5b, c). The reduction of MRTF-A and RhoA expression was more prominent in the UC-MSCs than in the PL-MSCs. To determine whether the UC/PL-MSCs affect the F-actin polymerization, which is downstream of the Rho/ROCK signaling and upstream of the MRTF-A/SRF signaling in the HIMFs, we stained the F-actin with rhodamine-phalloidin by immunocytochemistry. When compared with the controls, TGF-β1 enhanced the F-actin staining which demonstrates F-actin polymerization (stress fiber formation). Both the UC- and PL-MSCs reduced the TGF-β1-induced F-actin formation in the HIMFs (Fig. 6a). To further evaluate whether the inhibition of MRTF-A nuclear localization is also involved in the anti-fibrotic mechanism of the UC/PL-MSCs, we quantified the nuclear-to-cytoplasmic signal ratio in the MRTF-A immunocytochemistry. Both in unstimulated and TGF-β1-stimulated cells, the UC/PL-MSCs led to a significant reduction in MRTF-A nuclear localization (Fig. 6a, b). In summary, these data suggest that the UC/PL-MSCs may inhibit the Rho/MRTF/SRF signaling in the HIMFs.

### UC-MSCs decrease the TGF-β1-induced phosphorylation of Smad2 and Smad3

**Fig. 7:**
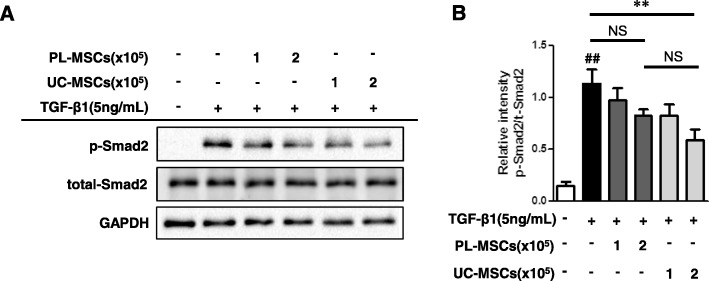
UC-MSCs inhibit TGF-β1-induced phosphorylation of Smad2. HIMFs were treated with TGF-β1 (5 ng/mL) and co-cultured with or without UC/PL-MSCs at 1 or 2 × 10^5^ cells/insert for 48 h. **a** Representative Western blots show the protein expression of phosphorylated Smad2 (p-Smad2) with total Smad2 and GAPDH as a loading control. **b** Quantitation of p-Smad2 from Western blot analyses (*n* = 4). Data are expressed as the means ± SEM. ^##^*P* < 0.01 versus the untreated control; ^**^P < 0.01 versus the TGF-β1 treatment only. NS, not significant

In addition, we examined whether the classical Smad-dependent TGF-β pathways [13] are implicated in the anti-fibrotic mechanism of the UC/PL-MSCs in HIMFs. As shown in Fig. 7, the UC-MSCs significantly decreased the Smad2 phosphorylation induced by the TGF-β1 treatment for 48 h in a dose-dependent manner. Though not statistically significant, the PL-MSCs also showed a reductive trend in the TGF-β1-induced phosphorylation of Smad2. To further confirm the inhibition of the Smad-dependent TGF-β pathways by the UC/PL-MSCs, we checked the phosphorylation of Smad2 and Smad3 at different time points (1 h and 24 h). At both time points, the UC-MSCs significantly decreased the TGF-β1-induced phosphorylation of Smad2 and Smad3. The PL-MSCs also showed a reductive trend but it was not statistically significant. These results suggest that the anti-fibrogenic actions of the UC-MSCs likely occur through Smad-dependent mechanisms (Additional file 3: Figure S3).

### Suppression of the ECM and α-SMA expression by the UC/PL-MSCs are independent of the phosphorylation of ERK, JNK, p38 MAPK, and AKT

We further evaluated to determine whether other Smad-independent TGF-β pathways are involved in the anti-fibrotic mechanism of the UC/PL-MSCs in the HIMFs. The HIMFs were pretreated with or without each of the four kinase inhibitors targeting ERK (U0126), JNK (SP600125), p38MAPK (SB203580), and AKT (LY294002) for 30 min prior to the addition of TGF-β1 (Additional file 4). As shown in Additional file 2: Figure S2A and B, each of the four kinase inhibitors significantly reduced the TGF-β1-induced ECM and α-SMA synthesis in a concentration-dependent manner. These findings, consistent with previous studies [8, 12, 14, 36], indicate that the TGF-β1-mediated ECM and α-SMA expression in the HIMFs occurred by the phosphorylation of non-Smad signaling molecules (ERK, JNK, p38MAPK, and AKT). However, none of the phosphorylated molecules were downregulated by the co-culture with the UC/PL-MSCs in the HIMFs activated by TGF-β1, suggesting that the UC/PL-MSCs may exert an anti-fibrotic effect without reducing the phosphorylation of ERK, JNK, p38MAPK, and AKT (Additional file 3: Figure S3).

## Discussion

We did an in vitro study demonstrating that two perinatal tissue-derived MSCs (UC- and PL-MSCs) inhibit the TGF-β1-induced fibrogenic activation of the HIMFs by reducing the expression of ECM (Procol1A1 and FN) and α-SMA. In addition, we confirmed that TGF-β1 induces ECM and α-SMA expression by activating the MRTF/SRF pathway as a Smad-independent TGF-β signaling mechanism in the HIMFs. Finally, we showed that the UC/PL-MSCs suppress TGF-β1-induced synthesis of ECM and α-SMA in the HIMFs by blocking Rho/MRTF/SRF signaling.

By evaluating the expression levels of the ECM proteins (Procol1A1, FN) and contractile protein (α-SMA), we showed that the co-culture with the UC/PL-MSCs significantly inhibited the TGF-β1-mediated fibrogenesis in the HIMFs. This is consistent with a previous report in which the co-culture with PL-MSCs reduced TGF-β1-induced collagen I synthesis in hepatic stellate cells [37]. UC-MSC-derived exosomes also decreased TGF-β-induced α-SMA expression in skin fibroblasts by the suppression of TGF-β/Smad2 pathway [38]. Besides myofibroblasts, the UC/PL-MSCs appear to inhibit fibrogenic gene expression in other types of cells [39–41]. UC-MSC-conditioned medium inhibited TGF-β1-induced α-SMA expression in renal tubular epithelial cells suggesting that the UC-MSCs exhibit their anti-fibrotic effect by inhibiting the epithelial-mesenchymal transition process which might provide a cellular source of myofibroblasts and contribute to fibrosis [40]. Recently, the treatment of PL-MSC-exosomes decreased the expressions of collagen 1A1 and collagen 1A2 in myoblasts derived from patients with Duchenne muscular dystrophy which is characterized by muscle fibrosis [39]. Consistent with these findings, previous animal studies have shown that UC/PL-MSCs alleviate fibrosis by reducing the expressions of collagen and α-SMA in various types of tissues including the liver [42, 43], pancreas [44], esophagus [45], lungs [46], kidneys [40], and skin [38]. In particular, some of those studies demonstrated that the transplantation of UC/PL-MSCs decreased the number of activated myofibroblasts (α-SMA positive) in the liver [42], pancreas [44], and esophagus [45], indicating that the UC/PL-MSCs inhibit the fibrogenic activation of myofibroblasts. Taken together, UC/PL-MSCs appear to inhibit fibrogenesis in myofibroblasts by reducing the expressions of ECM and α-SMA.

In our study, MRTF-A/SRF inhibitor (CCG-100602) diminished the TGF-β1-stimulated ECM and α-SMA expression in the HIMFs, which suggests that MRTF/SRF signaling may contribute to the fibrogenic activation of the HIMFs. The Rho/ROCK/Actin/MRTF/SRF signaling axis has been identified as a key pathway in multiple types of solid organ fibrosis [15, 47–50]. From the Rho family of GTPases, RhoA leads to the profilin-mediated incorporation of G-actin into F-actin (F-actin polymerization) by mDiaphanous activation. On the other hand, RhoA-mediated ROCK activation enables LIM kinase to inhibit cofilin-mediated depolymerization of F-actin [51]. F-actin polymerization releases G-actin-bound MRTF-A, which translocates to the nucleus and interacts with SRF, inducing fibrogenic genes including collagen 1A1, α-SMA, and myosin light chain kinase [10, 15]. Emerging evidence has revealed the role of Rho/ROCK/Actin/MRTF/SRF signaling in intestinal fibrosis [10, 52, 53]. Our findings are consistent with those from recent studies evaluating the role of Rho/MRTF/SRF signaling in intestinal fibrosis. For example, one report showed that novel Rho/MRTF/SRF pathway inhibitors (CCG-100602, CCG-203971), which specifically disrupt MRTF-A nuclear localization and consequently inhibit SRF transcription, repressed TGF-β1-induced collagen I and α-SMA expression in a human colonic myofibroblast cell line (CCD-18co) [10]. Recently, in human intestinal fibroblasts, ROCK inhibition reduced F-actin polymerization and, subsequently, prevented the TGF-β1-induced expression of collagen 1A1 and MRTF-A [52]. In addition, a previous study found that TGF-β1-induced Rho activation was more prominent in human smooth muscle cells, which were isolated from patients with radiation-induced ileal fibrosis, compared with normal ileum, suggesting that Rho signaling may operate during the chronic stage of intestinal fibrosis [53].

Although previous in vivo studies have shown that UC/PL-MSCs alleviate tissue fibrosis and several in vitro studies have demonstrated that UC/PL-MSCs prevent TGF-β1-induced fibrogenic activation in several types of cells including myofibroblasts, the intracellular and molecular mechanisms by which UC/PL-MSCs modulate TGF-β signaling pathways are poorly understood. In the present study, the co-culture with UC/PL-MSCs suppressed ECM and α-SMA expression in the HIMFs by attenuating the expressions of MRTF-A and SRF induced by TGF-β1 as well as by inhibiting the nuclear translocation of MRTF-A evident by the increased cytoplasmic staining. This result indicates that the UC/PL-MSCs exert an anti-fibrotic effect in the HIMFs by modulating the MRTF/SRF signaling. A previous study reported that the co-culture with hypoxia-preconditioned BM-MSCs inhibited collagen I and α-SMA synthesis in cardiac fibroblasts by reducing the expressions of MRTF-A and MRTF-B and by inhibiting the nuclear translocation of MRTF-A [54]. These findings suggest the possibility that the intracellular anti-fibrogenic action of MSCs may occur through a MRTF/SRF-dependent mechanism.

To further evaluate whether the UC/PL-MSCs modulate upstream molecules of the MRTF/SRF pathway, we checked the expression of RhoA, ROCK1, ROCK2, and F-actin in the HIMFs. Interestingly, the UC/PL-MSCs not only reduced the expression of MRTF-A and SRF but also suppressed the expression of RhoA, ROCK1, and ROCK2. In addition, the UC/PL-MSCs decreased TGF-β1-induced F-actin formation, as determined by the decreased F-actin fluorescent signal. Rho/ROCK signaling is an upstream pathway which mediates F-actin polymerization and subsequently MRTF/SRF transcription activation [15]. Therefore, UC/PL-MSCs may function more upstream in the TGF-β pathway, act more broadly than selective MRTF/SRF inhibition, and also interfere with the Rho/ROCK pathway, which subsequently results in F-actin depolymerization.

We further evaluated whether other Smad-independent TGF-β pathways and Smad-dependent TGF-β pathways are involved in the anti-fibrotic mechanism of the UC/PL-MSCs in the HIMFs. The UC/PL-MSCs failed to reduce the phosphorylation of ERK, JNK, p38 MAPK, and AKT. In contrast, the phosphorylated Smad2 and Smad3, which were induced in the HIMFs by TGF-β1 stimulation, were significantly reduced by the UC-MSCs but not by the PL-MSCs. These results indicate that the inhibition of the Smad-dependent TGF-β pathway may be another anti-fibrotic mechanism of the UC-MSCs. These findings are consistent with some prior studies, which reported that TGF-β/Smad2 signaling is involved in the anti-fibrotic mechanism of UC-MSCs [38, 41, 46]. In skin fibroblasts, UC-MSC-derived exosomes decreased TGF-β-induced α-SMA expression by the suppression of Smad2 activation [38]. In animal models of liver and lung fibrosis, UC-MSCs alleviated organ fibrosis and reduced the protein expression of phosphorylated Smad2 in whole organ protein extracts [41, 46].

Due to their ability to modulate fibrogenesis, MSCs are considered as a potential candidate for treating various fibrotic diseases [21]. Therefore, comparing the anti-fibrotic properties of MSCs derived from different sources may have great value in selecting the one which would be the most effective in clinical therapy. To determine which population of perinatal tissue-derived MSCs exhibits more prominent anti-fibrotic effects on the HIMFs, we compared MSCs derived from two different sources, the UC and PL. The inhibitory effects of the UC-MSCs on the TGF-β1-induced Procol1A1 and FN expression in the HIMFs were more prominent than those of the PL-MSCs. The UC-MSCs also reduced TGF-β1-induced α-SMA expression in the HIMFs to a higher degree compared to the PL-MSCs. These results suggest that the anti-fibrotic effects on the HIMFs might be more potent with the UC-MSCs than with the PL-MSCs. In our mechanism studies, the UC-MSCs exhibited more prominent inhibitory effects on the TGF-β1-induced protein expressions of MRTF-A, SRF, RhoA, and phosphorylated Smad2 and Smad3 compared with the PL-MSCs, which might support the mechanisms by which the UC-MSCs have a more potent anti-fibrotic activity than that of the PL-MSCs. In this regard, a previous study which compared MSCs derived from four different human sources (BM, AT, UC, and PL) showed that the UC-MSCs had the strongest immunosuppressive effects and the highest proliferative and differentiation potential [27]. Further in vivo studies are needed to confirm whether UC-MSCs will be more effective than PL-MSCs for the treatment of intestinal fibrosis.

## Conclusions

**Fig. 8:**
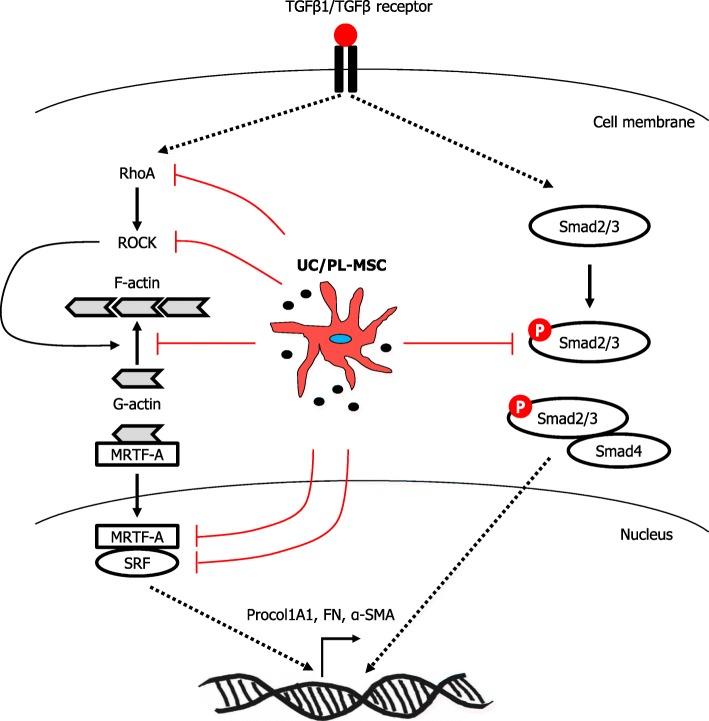
A proposed model showing the mechanism of action of UC/PL-MSCs on TGF-β1-induced fibrogenic activation in human primary intestinal myofibroblasts. TGF-β1 induces expression of Procol1A1, FN, and α-SMA by activating the MRTF-A/SRF signaling and Smad2/3. UC/PL-MSCs inhibit TGF-β1-induced fibrogenic responses by blocking the RhoA/MRTF-A/SRF signaling and Smad2/3

Based on our observations, we suggest that UC/PL-MSCs could prevent the TGF-β1-induced fibrogenic activation in the HIMFs by inhibiting the Rho/MRTF/SRF signaling (Fig. 8). The UC/PL-MSCs may represent a novel therapeutic strategy for intestinal fibrosis. However, further mechanistic and in vivo studies are needed to validate this novel finding.

## Additional files

Additional file 1: **Figure S1.** Characterization of UC/PL-MSCs. **(A, B):** The morphologies of UC-MSCs (A) and PL-MSCs (B) were similar to the round-spindle shape of mesenchymal stem cells (× 100). **(C, D):** FACS analysis of the expression of surface markers in UC-MSCs (C) and PL-MSCs (D). The percentages are indicated along with the fluorescence intensities. (PPTX 289 kb)

Additional file 2: **Figure S2.** TGF-β1-mediated fibrogenic action of HIMFs occurs by phosphorylation of ERK, JNK, p38MAPK, and AKT. HIMFs were pretreated with or without each of the 4 kinase inhibitors targeting ERK (U0126 at 5, 10, 20, and 40 μmol/L concentrations), JNK (SP600125 at 5, 10, 20, and 40 μmol/L concentrations), p38MAPK (SB203580 at 5, 10, 20, and 40 μmol/L concentrations), and AKT (LY294002 at 1, 5, 10, and 20 μmol/L concentrations) for 30 min prior to the addition of TGF-β1 (5 ng/mL) for 48 h. **(A):** Representative Western blots show the protein expression of Procol1A1, FN, and α-SMA with GAPDH as a loading control. **(B):** Quantitation of Procol1A1, FN, and α-SMA from the Western blot analyses (*n* = 3). Data are expressed as the means ± SEM. ^#^*P* < 0.05 versus the untreated control; ^*^P < 0.05, ^**^P < 0.01, and ^***^P < 0.001 versus the TGF-β1 treatment only. (PPTX 325 kb)

Additional file 3: **Figure S3.** The effects of UC/PL-MSCs in the TGF-β1-induced phosphorylation of Smad2, Smad3, ERK, JNK, p38MAPK, and AKT in HIMFs. HIMFs were treated with TGF-β1 (5 ng/mL) and co-cultured with or without UC/PL-MSCs at 2 × 10^5^ cells/insert for 1 (A, C) or 24 (B, D) hours. **(A, B):** Representative Western blots show the phosphorylated protein expression of Smad2, Smad3, ERK, JNK, p38MAPK, and AKT with GAPDH as a loading control. **(C, D):** Quantitation of the phosphorylated protein expression of Smad2, Smad3, ERK, JNK, p38MAPK, and AKT from the Western blot analyses (n = 3). Data are expressed as the means ± SEM. ^#^*P* < 0.05, ^##^*P* < 0.01 versus the untreated control; ^*^P < 0.05, ^**^P < 0.01, and ^***^P < 0.001 versus the TGF-β1 treatment only. (PPTX 153 kb)

Additional file 4: Supplementary Methods. (DOCX 13 kb)

## Data Availability

All data reported have been obtained from experiments carried out in the author’s laboratory. The dataset generated during the present study is available upon reasonable request to the corresponding authors (Prof. Jun-Hwan Yoo or Prof. Duk Hwan Kim).

## Abbreviations

ANOVA: Analysis of variance
AT: Adipose tissue
BM: Bone marrow
CD: Crohn’s disease
ERK: Extracellular signal-regulated kinase
ECM: Extracellular matrix
F-actin: Filamentous actin
FN: Fibronectin
G-actin: Globular-actin
GAPDH: Glyceraldehyde-3-phosphate dehydrogenase
GCP: Good clinical practice
GMP: Good manufacturing practices
HIMFs: Human primary intestinal myofibroblasts
IBD: Inflammatory bowel diseases
JNK: c-Jun N-terminal kinase
MAPK: Mitogen-activated protein kinase
MRTF-A: Myocardin-related transcription factor A
MSCs: Mesenchymal stem cells
Procol1A1: Procollagen1A1
RhoA: Ras homolog gene family, member A
ROCK: Rho-associated coiled-coil forming protein kinase
SEM: Standard error of the mean
SRF: Serum response factor
TGF-β: Transforming growth factor-beta
α-SMA: α-smooth muscle actin
