# Supplementary figures and images for: Umbilical cord/placenta-derived mesenchymal stem cells inhibit fibrogenic activation in human intestinal myofibroblasts via inhibition of myocardin-related transcription factor A
Source: Stem Cell Res Ther. 2019 Sep 23;10:291. doi: 10.1186/s13287-019-1385-8 (PMC6757442; doi:10.1186/s13287-019-1385-8)

## Slide 1
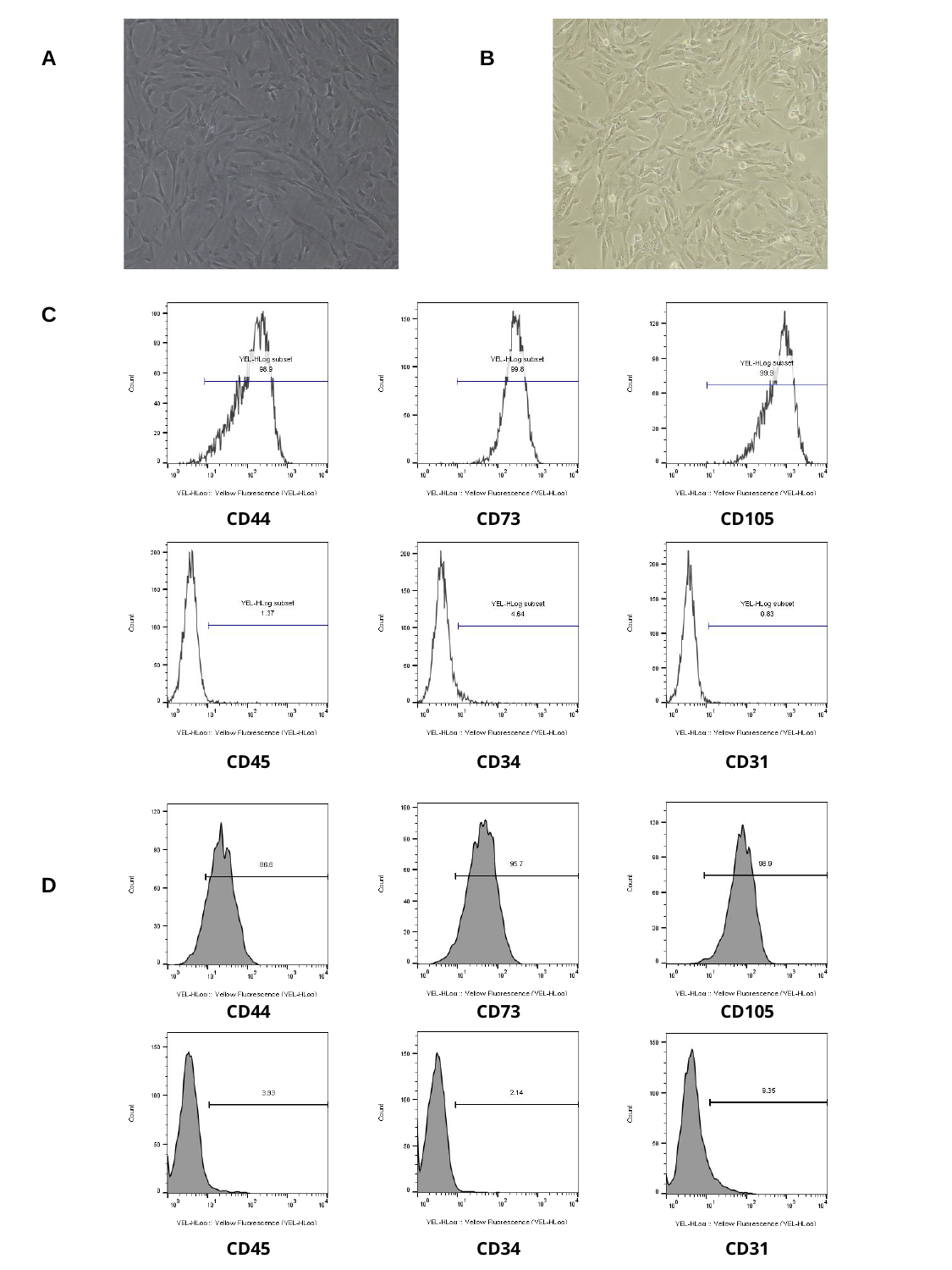

A
B
C
CD44
CD73
CD105
CD45
CD34
CD31
D
CD44
CD73
CD105
CD45
CD34
CD31

Supplement: Supplementary file 1 — Figure S1. Characterization of UC/PL-MSCs. (A, B): The morphologies of UC-MSCs (A) and PL-MSCs (B) were similar to the round-spindle shape of mesenchymal stem cells (× 100). (C, D): FACS analysis of the expression of surface markers in UC-MSCs (C) and PL-MSCs (D). The percentages are indicated along with the fluorescence intensities. (PPTX 289 kb) [file 13287_2019_1385_MOESM1_ESM.pptx]
