# Supplementary material for: Umbilical cord/placenta-derived mesenchymal stem cells inhibit fibrogenic activation in human intestinal myofibroblasts via inhibition of myocardin-related transcription factor A
Source: Stem Cell Res Ther. 2019 Sep 23;10:291. doi: 10.1186/s13287-019-1385-8 (PMC6757442; doi:10.1186/s13287-019-1385-8)
Supplement: Supplementary file 2 — Figure S2. TGF-β1-mediated fibrogenic action of HIMFs occurs by phosphorylation of ERK, JNK, p38MAPK, and AKT. HIMFs were pretreated with or without each of the 4 kinase inhibitors targeting ERK (U0126 at 5, 10, 20, and 40 μmol/L concentrations), JNK (SP600125 at 5, 10, 20, and 40 μmol/L concentrations), p38MAPK (SB203580 at 5, 10, 20, and 40 μmol/L concentrations), and AKT (LY294002 at 1, 5, 10, and 20 μmol/L concentrations) for 30 min prior to the addition of TGF-β1 (5 ng/mL) for 48 h. (A): Representative Western blots show the protein expression of Procol1A1, FN, and α-SMA with GAPDH as a loading control. (B): Quantitation of Procol1A1, FN, and α-SMA from the Western blot analyses (n = 3). Data are expressed as the means ± SEM. #P < 0.05 versus the untreated control; *P < 0.05, **P < 0.01, and ***P < 0.001 versus the TGF-β1 treatment only. (PPTX 325 kb) [file 13287_2019_1385_MOESM2_ESM.pptx]

## Slide 1
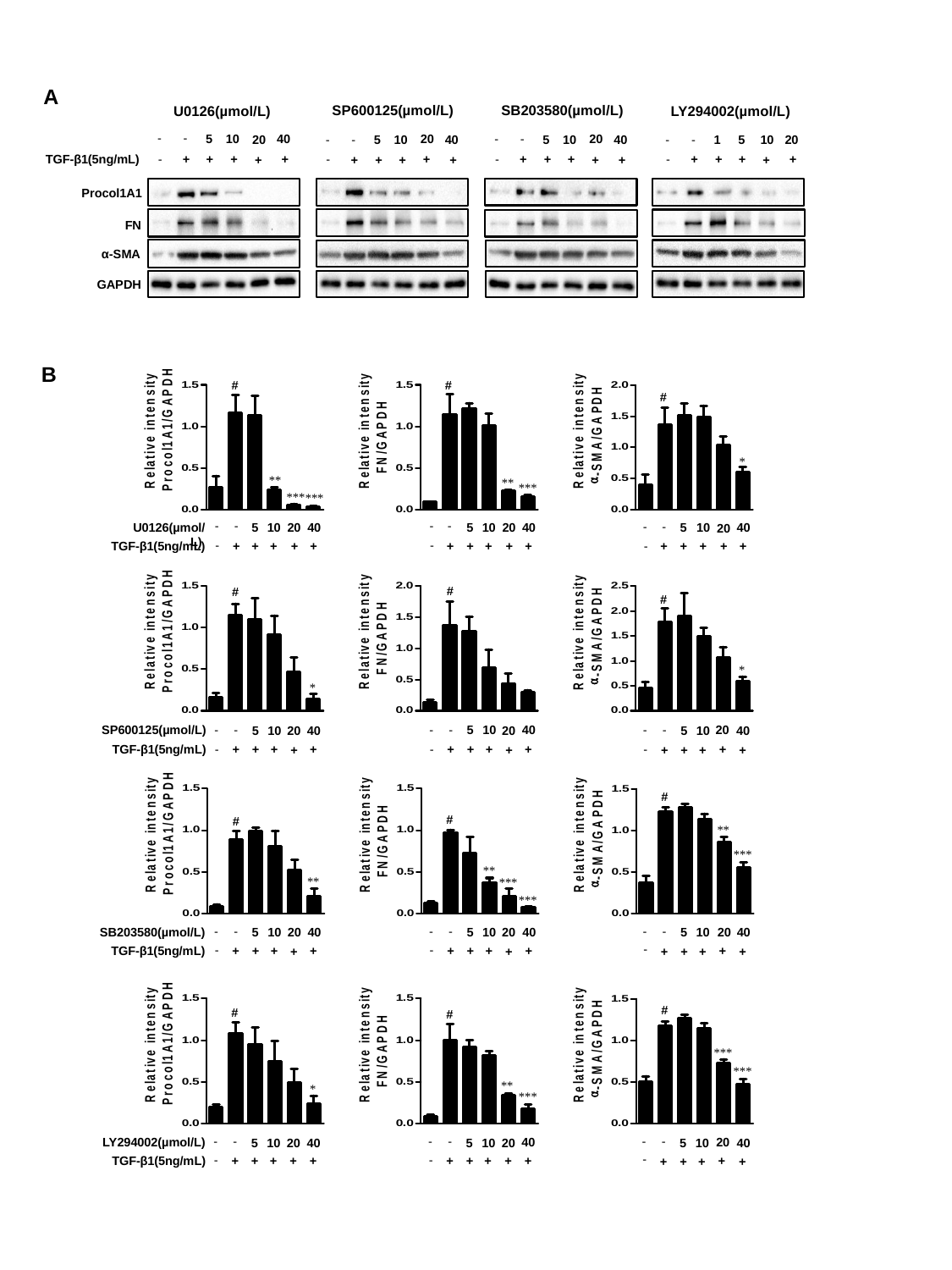

A
SP600125(µmol/L)
SB203580(µmol/L)
LY294002(µmol/L)
U0126(µmol/L)
-
-
5
10
40
-
-
20
20
20
1
5
20
5
10
40
10
5
10
40
-
-
-
-
-
-
TGF-β1(5ng/mL)
+
+
+
+
-
-
+
+
+
+
+
+
+
+
+
+
+
+
+
+
+
+
Procol1A1
FN
α-SMA
GAPDH
B
#
#
#
*
**
**
***
***
***
-
-
-
-
-
-
U0126(µmol/L)
20
20
5
10
40
5
10
40
5
10
40
20
-
-
+
+
+
-
+
+
+
+
+
+
+
+
+
+
+
TGF-β1(5ng/mL)
+
#
#
#
*
*
-
-
-
-
-
-
SP600125(µmol/L)
20
5
10
40
5
10
40
20
5
10
40
20
-
-
TGF-β1(5ng/mL)
-
+
+
+
+
+
+
+
+
+
+
+
+
+
+
+
#
#
#
**
***
**
**
***
***
-
-
-
-
-
-
SB203580(µmol/L)
20
5
10
40
20
20
5
10
40
5
10
40
-
-
-
TGF-β1(5ng/mL)
+
+
+
+
+
+
+
+
+
+
+
+
+
+
+
#
#
#
***
***
**
*
***
-
-
-
-
-
-
LY294002(µmol/L)
20
40
40
5
10
5
10
20
5
10
40
20
-
-
-
+
+
+
TGF-β1(5ng/mL)
+
+
+
+
+
+
+
+
+
+
+
+
