# Supplementary material for: Umbilical cord/placenta-derived mesenchymal stem cells inhibit fibrogenic activation in human intestinal myofibroblasts via inhibition of myocardin-related transcription factor A
Source: Stem Cell Res Ther. 2019 Sep 23;10:291. doi: 10.1186/s13287-019-1385-8 (PMC6757442; doi:10.1186/s13287-019-1385-8)
Supplement: Supplementary file 3 — Figure S3. The effects of UC/PL-MSCs in the TGF-β1-induced phosphorylation of Smad2, Smad3, ERK, JNK, p38MAPK, and AKT in HIMFs. HIMFs were treated with TGF-β1 (5 ng/mL) and co-cultured with or without UC/PL-MSCs at 2 × 10^5^ cells/insert for 1 (A, C) or 24 (B, D) hours. (A, B): Representative Western blots show the phosphorylated protein expression of Smad2, Smad3, ERK, JNK, p38MAPK, and AKT with GAPDH as a loading control. (C, D): Quantitation of the phosphorylated protein expression of Smad2, Smad3, ERK, JNK, p38MAPK, and AKT from the Western blot analyses (n = 3). Data are expressed as the means ± SEM. #P < 0.05, ##P < 0.01 versus the untreated control; *P < 0.05, **P < 0.01, and ***P < 0.001 versus the TGF-β1 treatment only. (PPTX 153 kb) [file 13287_2019_1385_MOESM3_ESM.pptx]

## Slide 1
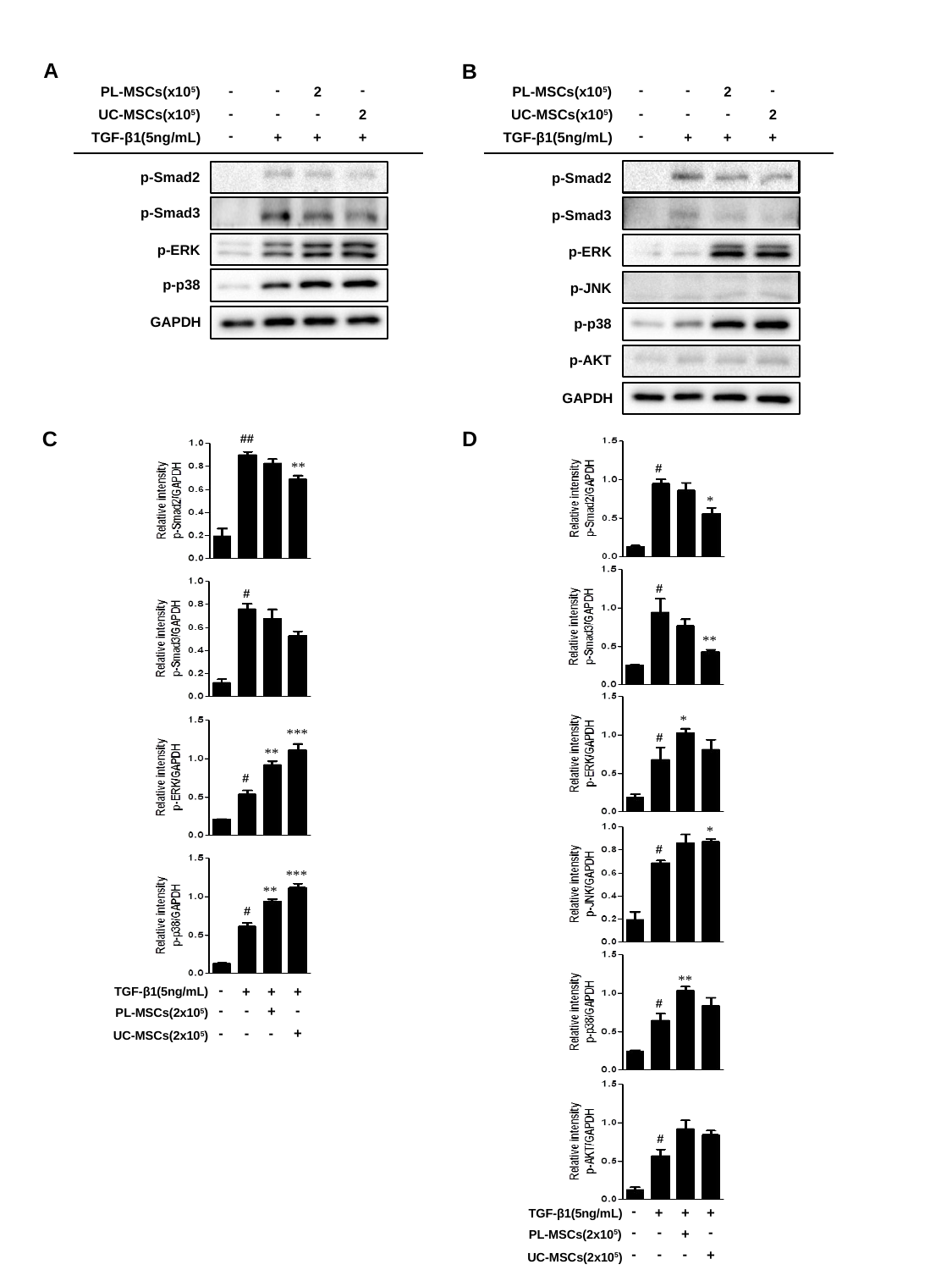

A
B
-
-
-
-
-
-
PL-MSCs(x105)
PL-MSCs(x105)
2
2
-
-
-
-
-
-
UC-MSCs(x105)
2
UC-MSCs(x105)
2
-
-
TGF-β1(5ng/mL)
TGF-β1(5ng/mL)
+
+
+
+
+
+
p-Smad2
p-Smad2
p-Smad3
p-Smad3
p-ERK
p-ERK
p-p38
p-JNK
GAPDH
p-p38
p-AKT
GAPDH
D
C
##
**
#
*
#
#
***
#
**
#
#
***
**
#
-
+
+
+
TGF-β1(5ng/mL)
#
-
-
-
+
PL-MSCs(2x10^5^)
-
-
-
+
UC-MSCs(2x10^5^)
#
-
+
+
+
TGF-β1(5ng/mL)
-
-
-
+
PL-MSCs(2x10^5^)
-
-
-
+
UC-MSCs(2x10^5^)
**
*
*
**
