# Supplementary material for: Umbilical cord/placenta-derived mesenchymal stem cells inhibit fibrogenic activation in human intestinal myofibroblasts via inhibition of myocardin-related transcription factor A
Source: Stem Cell Res Ther. 2019 Sep 23;10:291. doi: 10.1186/s13287-019-1385-8 (PMC6757442; doi:10.1186/s13287-019-1385-8)
Supplement: Supplementary file 4 — Supplementary Methods. (DOCX 13 kb) [file 13287_2019_1385_MOESM4_ESM.docx]

**Supplementary Methods**

**Kinase inhibitor treatment**

To block the other Smad-independent TGF-β pathways, we used an ERK inhibitor (U0126), a JNK inhibitor (SP600125), a p38MAPK inhibitor (SB203580), and an AKT inhibitor (LY294002). All the inhibitors were obtained from Calbiochem (San Diego, CA). The HIMFs were seeded at a density of 1.5 × 10^5^ cells/well in 6-well plates. Dose dependence for each inhibitor was determined by pretreating cells in serum-free medium for 30 minutes, after which 5 ng/mL of TGF-β1 were added to the medium, and the cells were incubated for 48 hours. Western blots were performed to assess the expression of Procol1A1, FN and α-SMA as described earlier.
